# Pattern‐Aware Intelligence Enables Nondestructive, Rapid Quantification of High‐Aspect‐Ratio Silicon Etching

**DOI:** 10.1002/advs.76723

**Published:** 2026-07-20

**Authors:** Shuyan He, Lang Chen, Bo Wen, Yufeng Jin, Wei Wang

**Affiliations:** ^1^ School of Integrated Circuits Peking University Beijing China; ^2^ School of Electronic and Computer Engineering Peking University Beijing China; ^3^ National Key Laboratory of Advanced Micro and Nano Manufacture Technology Beijing China; ^4^ Beijing Advanced Innovation Center for Integrated Circuits Beijing China

**Keywords:** high‐aspect‐ratio structure, microelectromechanical system fabrication, physics‐informed machine learning, virtual metrology

## Abstract

High‐aspect‐ratio (HAR) structures are widely used in microelectromechanical systems (MEMS), electronic devices, and advanced packaging. Accurate characterization of post‐etch microstructural morphology is essential for process control and device reliability. However, existing metrology techniques rely on destructive methods and cannot capture the complete three‐dimensional profile. This study proposes a pattern‐aware intelligent model that transforms pattern‐dependent etching behavior into a physical prior, enabling rapid and minimally destructive reconstruction of HAR structures across pattern scales. A YOLO‐Pose‐based model is developed to automatically extract subpixel geometric features from scanning electron microscopy images, achieving a quantification accuracy of 95.11% with *R* ^2^ >0.98 for key dimensions. By incorporating the pattern‐dependent effect in HAR silicon etching as a physical prior, the model creates a topography network that captures etch behavior across feature sizes under identical process conditions. With minimal destructive observations of shallow regions of the wafer, the framework achieves a 93.96% prediction accuracy in the nondestructive retrieval of critical parameters, including etch depth, sidewall angle, and scallop texture, while demonstrating robust cross‐scale generalization across varying layout densities. This approach reduces the data acquisition time to 1 min, enabling near‐real‐time process feedback. It provides a highly interpretable, cost‐effective, and intelligent solution for intelligent manufacturing applications.

## Graphical abstract

Pattern‐dependent etching is converted into a physical prior for intelligent reconstruction of high‐aspect‐ratio silicon structures. Combining YOLO‐Pose feature extraction with a topography network, the framework retrieves depth, sidewall angle, and scallop texture from minimal destructive observations, enabling accurate cross‐scale metrology and near‐real‐time feedback for high‐aspect‐ratio microfabrication.

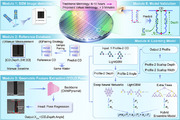

## 1 Introduction

As the global semiconductor industry enters the post‐Moore's law era [1, 2, 3] further enhancements in chip performance and functionality become increasingly reliant on advances in three‐dimensional (3D) structures and silicon‐based micro‐ and nanofabrication technologies [4, 5, 6, 7]. As a cornerstone process, deep reactive ion etching (DRIE) broadly supports the realization of key interconnect structures, such as functional microstructures in microelectromechanical system (MEMS) sensors [8, 9, 10] high‐aspect‐ratio (HAR) trench isolation in power/radio‐frequency (RF) devices [11, 12], and through‐silicon vias (TSVs) and silicon‐based interposers in advanced packaging [13, 14].

Unlike shallow etching in conventional front‐end processes, ion incidence, reactant supply, and byproduct removal in HAR channels are constrained by geometry [15, 16]. Combined with large‐area exposure and localized aperture ratio variations, these constraints significantly amplify pattern‐dependent effects (PDEs), such as the local area loading (LAG) effect [17, 18]. Under identical plasma conditions, patterns with varying widths and packing densities exhibit markedly different etch rates and topographies owing to significant disparities in ion fluxes and neutral‐species transport efficiency [19]^1^. This uncertainty in cross‐sectional morphology directly translates into a variation in key performance metrics across multiple device categories. For example, the TSV sidewall profile negatively impacts high‐frequency interconnects, as surface roughness significantly contributes to conductor loss by lengthening current pathways and increasing scattering at the substrate–conductor interface [20].

In power devices, serrations or damage to trench sidewalls can induce gate‐oxide nonuniformity and gate–source leakage [21, 22]. In MEMS silicon‐based microfluidic channels, DRIE sidewall roughness can reach a root‐mean square of approximately 0.081 µm, exhibiting quantifiable flow resistance characteristics under a Reynolds number of 0.38–0.60 and pressure drops of 17.2–19.5 kPa. Furthermore, studies have indicated that when the relative roughness reaches ∼2%, the pressure drop increases by 3.0–5.9% [23, 24, 25] Therefore, establishing rapid, nondestructive, and quantifiable methods for observing and providing feedback on cross‐sectional morphology of HAR structures formed by silicon etching can substantially reduce the metrology cost and cycle time while minimizing the wafer loss, thereby accelerating the yield ramp‐up and improving manufacturing sustainability in advanced semiconductor production.

However, morphological characterization is highly dependent on destructive cutting and scanning electron microscopy (SEM) observations [26, 27]. Furthermore, this process is expensive, inefficient, and limited in sampling coverage. More critically, its destructive nature renders wafers unusable for subsequent manufacturing steps, resulting in the irreversible loss of experimental samples. Focused ion beam (FIB) technology [28, 29], which initially provided an in‐line localized cross‐section observation method and gradually evolved into a key on‐chip inspection approach, exhibits a strong correlation among the cutting time, etch depth, and processed area. For HAR structures extending hundreds of micrometers in depth, preparing a single cross‐section sample may take several hours, severely limiting the application efficiency in production‐line environments. Furthermore, novel inspection methods, such as optical measurements [30, 31, 32] (e.g., spectroscopic ellipsometers and scattering measurements), acoustic imaging (scanning acoustic microscopy) [33, 34], and indirect electrical structure measurements [35, 36], have potential for large‐scale, nondestructive inspections.

However, these methods typically excel at measuring “indirect observables,” such as the film thickness, average geometry, or defect indications. They exhibit limited sensitivity to the local topographical details of HAR structures, including angular variations, bottom topography, and sidewall scalloping, and rely heavily on model assumptions, prior structural parameters, and calibration data. In practical chip layouts characterized by diverse pattern types, wide aperture ranges, and complex layout contexts, these methods often struggle to stably and accurately reconstruct complete 3D cross‐sectional profiles. Therefore, nondestructive, high‐throughput online observation of deep silicon etching topography is critical for enabling rapid process optimization, yield scaling, and manufacturing consistency across electronic devices, MEMS sensors, and advanced packaging technologies.

This study proposes an artificial intelligence (AI) model for the virtual measurement (VM) of etching topography that integrates data‐driven approaches with physical mechanisms. The method leverages the inherent PDE of HAR silicon etching as a physical prior. It constructs a topography mapping network for multiscale patterns using the same etching recipe. Using only minimal destructive observations of shallow wafer regions, it enables nondestructive, automated extraction of topography parameters across HAR areas throughout the wafer. Building on the YOLO‐Pose architecture, a regression model was constructed using a deep convolutional neural network (CNN). Through end‐to‐end feature training and inference, this model automatically identifies subpixel geometric keypoints and calculates the corresponding parameters from massive SEM images. The physical dimension measurement results achieved >90% agreement with the manual SEM measurements, with MAP50‐95 metrics exceeding 90%. For morphology modeling and prediction, a cascade model and hybrid ensemble model combining decision trees and neural networks were developed to address the high cost of acquiring internal morphology data in HAR etching and the challenges of fitting the nonlinearities in Bosch processes. Guided by the physical characteristics of deep silicon etching and constrained by limited historical morphology data, these models learn a mapping from surface‐observable features to the bottom morphology based on the top‐opening dimensions. This mapping enables the nondestructive inversion of key buried parameters, such as the etch depth, sidewall inclination, and microscopic scalloping.

## 2 Results

### 2.1 Model Architecture

**FIGURE 1:**
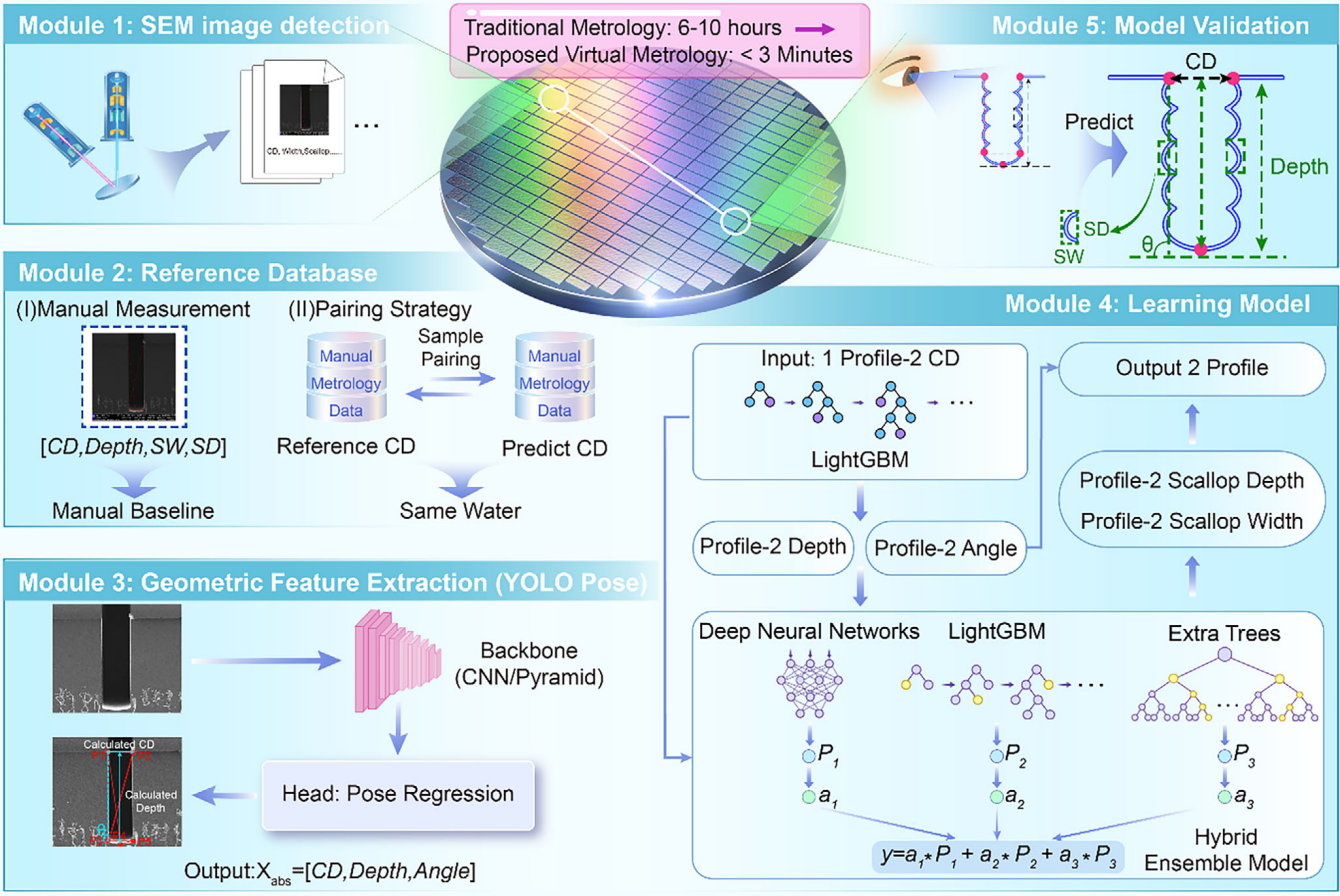
Schematic of the pattern‐aware intelligent model for HAR profile prediction.

Figure 1 illustrates the complete architecture of the proposed automated image feature extraction and VM models. Module 1 acquires raw cross‐sectional data via SEM imaging. Module 2 converts image data to manually measured textual data, which serve as the baseline for the “visual encoder.” It employs a “pairing strategy” to match etched structures with different aperture widths within the same wafer, thereby constructing the database for the subsequent prediction model. The data are then fed into Module 3 (Geometric Feature Extraction), where the proposed YOLO‐Pose deep learning network serves as the visual encoder. The encoder automatically extracts absolute feature vectors *X_abs_* =  [*CD*,  *Depth*,  *Angle*], where *CD* represents the channel depth, from unstructured SEM images with subpixel precision.

Module 4 employs a physics‐based multidimensional cascaded architecture. This model takes physical parameters extracted from the etching‐paired structures of the preceding module as inputs, distinguishing between reference and predicted etches. It uses the morphological physical parameters of the reference etch profile [*CD*,  *Depth*,  *Angle*,  *SW*,  *SD*], where *SW* and *SD* represent the scallop width and depth, respectively, to infer the corresponding buried morphology of the predicted etch. A prediction model for the etch depth (*Depth*) and sidewall angle (*Angle*) in the reference etch profile is established using Light Gradient‐Boosting Machine (LightGBM), incorporating physical constraints, such as the lag effect, to effectively expand the feature dimensionality. Subsequently, to analyze the etch sidewall microstructures (scallops), the obtained prediction results (*Width* and *Angle* from the reference etch profile) along with the morphological physical parameters [*CD*,  *Depth*,  *Angle*,  *SW*,  *SD*] and the *CD* value from the reference etch profile are used in the next stage. A hybrid ensemble strategy is implemented to construct an integrated model combining three distinct algorithms: a deep neural network (DNN) for high‐dimensional feature abstraction, LightGBM for gradient‐based decision‐making, and Extreme Random Trees (Extra Trees) to reduce the variance through randomness. The final morphological output is synthesized through a weighted‐ensemble mechanism $\hat{y}=\sum_{i=1}^{N}P_{i}\cdot a_{i}\left(x\right)$, where adaptive weights *P_i_* dynamically balance the contribution of each submodel and *a_i_* (*x*) represents the result obtained by each algorithm. This ensemble model ensures robust prediction accuracy across datasets of varying quality.

Finally, Module 5 (Model Validation) reconstructs the complete 3D etching profile. By outputting a full set of topography parameters, the system achieves comprehensive “virtual metrology” capabilities, enabling engineers to rapidly visualize the topography of etched structures. This AI‐driven virtual metrology approach, which automatically extracts image features using visual models and leverages pattern‐dependent effects, significantly reduces the measurement time to <3 min. In contrast to traditional destructive slicing methods, which typically require 6–10 h, the proposed method delivers near‐lossless, real‐time feedback for HAR etching processes.

### 2.2 Image Feature Extraction

**FIGURE 2:**
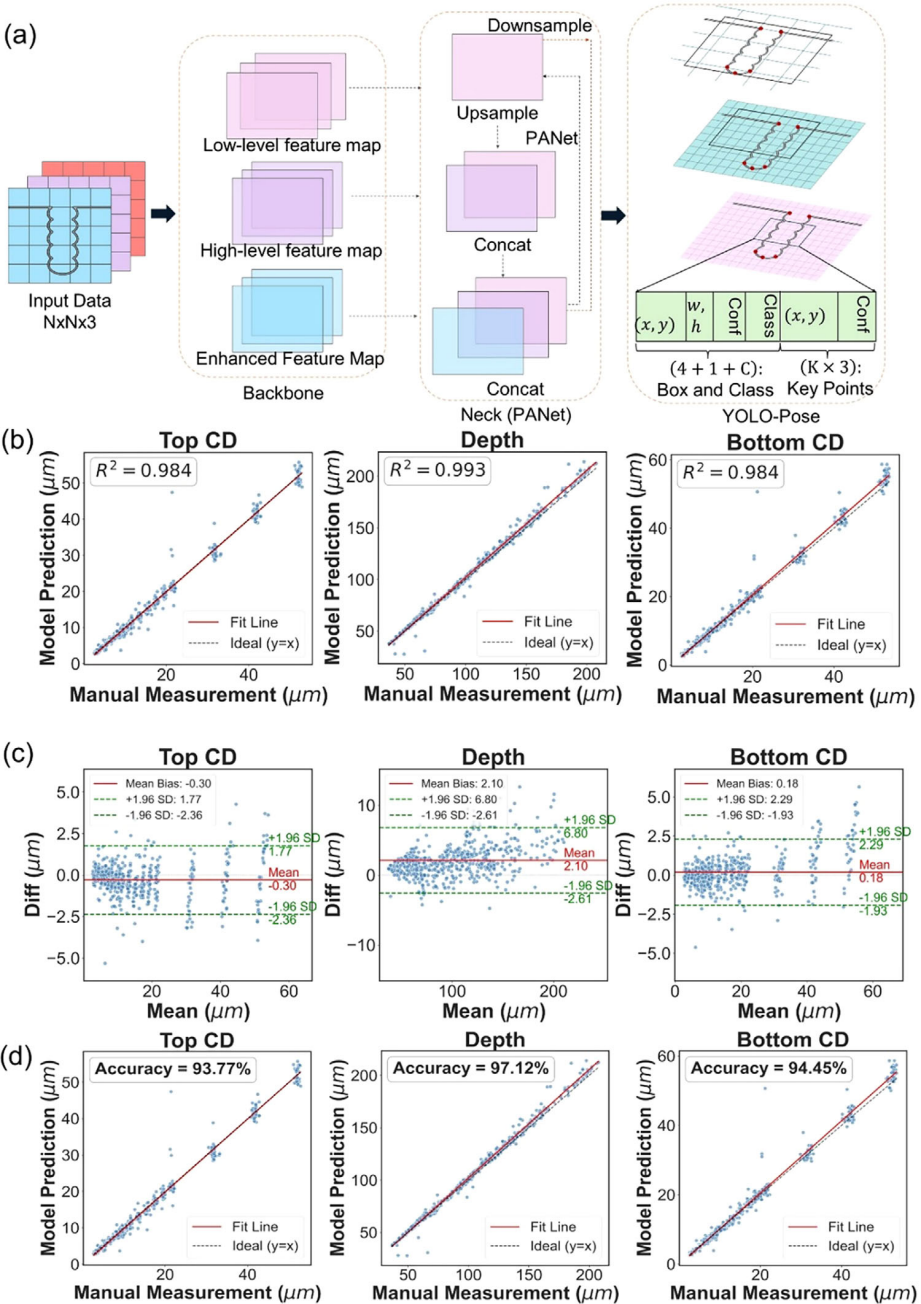
YOLO‐Pose structural diagram and image feature extraction results: (a) schematic of the YOLO‐Pose framework; (b) correlation coefficient for the *Depth* and *CD*; (c) Bland–Altman plot for the *Depth* and *CD*; (d) accuracy of *Depth* and *CD* in the validation set.

To enable automated batch data extraction from SEM test patterns, a computer‐vision‐based morphology extraction model was developed. The model architecture shown in Figure 2a processes the input SEM images of the etched interfaces using a dedicated deep CNN based on YOLO‐Pose. The backbone network serves as a feature extractor, generating hierarchical feature maps ranging from low‐level texture details to high‐level semantic abstractions. To address the significant scale variations observed in silicon‐etching SEM images, a Path Aggregation Network (PANet) is employed as the network neck. This component facilitates multiscale feature fusion through bidirectional upsampling and stitching. This design ensures that the model is sensitive to both global structural context and local edge nuances. The detection head subsequently outputs a composite vector containing the bounding‐box center coordinates, geometric features of the bounding box, class probabilities, and geometric keypoints for each pose point (*K* × 3), comprising point coordinates and confidence scores. By calculating the coordinates of these keypoints, the physical coordinates of the top‐opening width *w_top_*, bottom width *w_bottom_*, and *Depth* of the silicon‐etching SEM image are derived, enabling end‐to‐end subpixel‐level physical dimension regression directly from the raw image data. The sidewall tilt angle θ is calculated as follows:

(1) $$\theta =\arctan\left(\frac{Depth}{\frac{w_{top}-w_{bottom}}{2}}\right)\;\times \frac{180}{\pi }$$

To establish a robust ground truth for this supervised learning task, a statistically representative subset (153 groups, accounting for 21.73% of the total dataset) was manually annotated using the Labelme software. Compared with traditional bounding‐box detection, more precise five‐point annotations were employed to accurately define the geometric features of the etched structures, thereby establishing an intelligent data extraction model. The validation‐set regression analysis of the key silicon etching morphology parameters (*Depth*, *w_top_*, and *w_bottom_*) based on the YOLO‐Pose (YOLOv11) architecture is shown in Figure 2b. The model demonstrated exceptional feature extraction and localization capabilities, achieving Pose mAP50‐95 of 0.995 and Box mAP50‐95 of 0.969. These results confirm the ability of the algorithm to precisely pinpoint submicrometer‐scale geometric keypoints rather than merely providing coarse bounding boxes around the etched regions. Regarding the fitting accuracy, the coefficient of determination *R* ^2^ for the *Depth* reached 0.993, whereas *R* ^2^ for the *w_top_* and *w_bottom_* was 0.984. These results indicated that the model captured >98% of the physical topography variance, enabling high‐fidelity extraction from the image representation to automated physical data. The Bland–Altman analysis in Figure 2c validates the metrological reliability of the proposed method. The difference plot shows that the differences are tightly clustered around the mean bias line with no discernible divergence trend, indicating negligible systematic error. The mean biases for *w_top_*, *Depth*, and *w_bottom_* are controlled at −0.30, 2.10, and 0.18 µm, respectively. Moreover, most data points fall within the 95% limits of agreement (±1.96 SD), confirming the outstanding robustness and low uncertainty of the automated measurement method.

Based on the aforementioned intelligent data extraction model, the remaining 78.27% of the total data subset (551 groups) were tested and compared with manual SEM measurements. The manual measurements served as the baseline, whereas the model‐extracted values were used to calculate the errors. As shown in Figure 2d, the accuracy rates for the *Depth*, *w_top_*, and *w_bottom_* were 97.12%, 94.45%, and 93.77%, respectively. As *Angle* varied within a narrow range of approximately 89.5°–90°, it was not evaluated separately for assessing the performance of the YOLO model. However, the prediction accuracy for the *Angle* was 98.99%.

### 2.3 Feature Dimension Reduction

**FIGURE 3:**
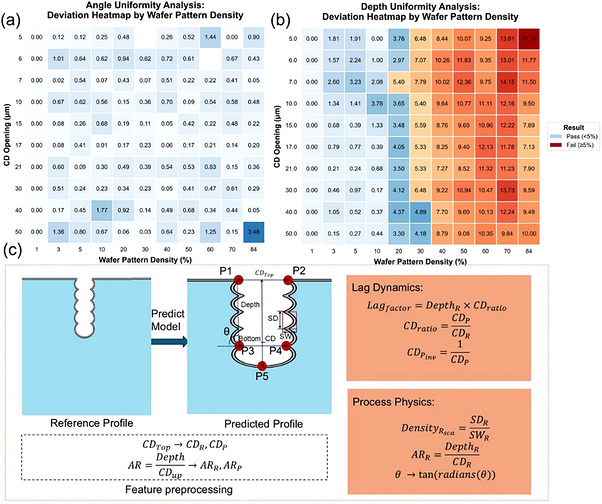
Pattern dependency analysis and model dimension expansion treatment: (a) effect of the pattern density on *Angle* in deep silicon etching; (b) effect of pattern density on *Depth* in deep silicon etching; (c) partial parameter display for establishing model feature dimensions.

Variations in pattern density significantly affected the final morphology of the etched patterns. To quantitatively describe the relationship between the pattern density and etching morphology, a pattern‐density gradient ranging from 1% to 84% across an 8‐inch (200‐mm) wafer was designed. Within this density range, patterns with different linewidths were selected for quantitative analysis. Using 1% pattern density as the baseline reference, the impact of pattern‐density variations on the etching morphology of patterns with identical linewidths was characterized. Figure 3a,b present the deviation heatmaps for patterns of the same linewidth under different pattern densities obtained through comparative calculations. Figure 3a demonstrates significant robustness of the sidewall angle to the pattern density, with angular deviation consistently suppressed below the 5% threshold across nearly all *CD* apertures (5–50 µm) and varying pattern densities. This indicates that the ion directionality is primarily governed by the sheath potential rather than by the reactant transport kinetics. Figure 3b shows the nonlinear relationship in the *Depth* uniformity. A distinct inflection point was observed at a pattern density of approximately 20%. Below this threshold, the etch‐depth variation due to the density remained consistently within 5%. Given the inherent 5% wafer uniformity error in the etch tool, variations below 5% were negligible for the given pattern density. However, when the pattern density exceeded 20%, features with different *CD* were significantly affected by the density. In extreme cases (e.g., 84% density), the *Depth*deviation increased to >17%, far exceeding the process tolerance. Considering this nonlinear, density‐dependent transfer phenomenon, the subsequently established predictive model is applicable only to pattern densities of <20%, at which pattern‐dependent effects remain weak.

To better describe the physical relationships among the etching morphology parameters in Figure 3c and explicitly incorporate nonlinear transport kinetics into the predictive inference, a high‐dimensional feature engineering framework was developed for mathematically characterizing these complex physical interactions. To transform the geometric “reference contour” into a model with physical predictive capability, 22 feature dimensions were extracted, as listed in Table S1. Figure 2c highlights seven decisive physical descriptors. In contrast to traditional black‐box data‐driven approaches, this feature space explicitly integrates the domain knowledge governing the etch rate and morphology quality. Seven representative physical descriptors are schematically shown. To account for the reactive ion etching (RIE) lag mechanism ($Lag_{factor},\;CD_{ratio},\;CD_{P_{inv}}$), the model explicitly incorporates the aspect‐ratio‐dependent etching (ARDE) by introducing *Lag_factor_* and *CD_ratio_*, thereby directly quantifying the sensitivity of RIE lag effects to variations in *Depth* relative to *CD*. The design of $CD_{P_{inv}}$ is based on the Knudsen diffusion theory: the gas‐transport resistance in a narrow channel is inversely proportional to the diameter. This inverse term successfully linearizes the transport resistance, which increases sharply and nonlinearly as *CD* decreases, reflecting the etch‐rate saturation due to the neutral‐particle‐flux limitation. This behavior corresponds to the manifestation of the RIE lag effect in the etching results.

Furthermore, a set of process physics descriptors ($Density_{R_{sca}},\;AR_{R},\;\tan\left(\theta \right)$) is introduced for parameter preprocessing during model construction. $Density_{R_{sca}}$ reveals the microscopic sidewall roughness produced by the alternating cycles in the Bosch process by analyzing the geometric proportions of the scallop. *AR_R_*, a critical characteristic of deep silicon etching, represents the aspect ratio of the reference etch. For sidewalls with extremely high verticality (typically distributed within the narrow range of 88.5°–90°), a tangent transformation (θ→tan(θ)) is applied to the angular feature. This nonlinear amplification mechanism maps minute angular deviations to a vast numerical space ([38.19, +∞]), significantly enhancing the numerical sensitivity of the model to subtle variations in the inclination of vertical sidewalls.

### 2.4 Morphological Prediction Model

To achieve the transition from discrete observation to predictive synthesis, a comprehensive dataset comprising 15 000 wafer‐sample pairs was constructed through initial data cleansing and a rigorous pairing strategy. The data cleansing involved excluding etched structures with obvious physical inconsistencies, primarily by limiting *Angle* to 85°–90° and enforcing *SW* and *SD* values within 3 µm. The pairing strategy restricted matching to structures within the same wafer and paired etched features with different *CD* values. The paired etch structures were categorized as either reference or predicted etch, incorporating the standard geometric attributes (*CD_R_*, *Depth_R_*, *Angle_R_*, *SW_R_*, *SD_R_*) of the reference etch along with the opening width (*CD_P_*) of the predicted etch. However, raw geometric scalars alone are insufficient to characterize complex transport kinetics. Therefore, in the Input Data module (Step 2), a physics‐informed bridge was constructed between the paired geometry and predictive models by expanding the feature space. Lag dynamics features (quantifying the transmission dependent on the aspect ratio) and process physics features (describing passivation/etching mechanisms in the Bosch cycle) were incorporated to capture the nonlinear control equations of the etching process. The detailed procedure and the full feature dimensions are shown in Figure 3c.

Given the strong correlation between scallop‐pattern formation and the depth and angle of the current etching structure, a hierarchical cascading architecture was designed. The model first employs LightGBM to predict the primary contour parameters: etch depth and sidewall angle (*Depth_P_*, *Angle_P_*). LightGBM was selected for its efficiency and accuracy in handling nonlinear gradients. The model achieved a preliminary accuracy of >95% for depth prediction. For angle prediction, the raw data underwent tangent‐function preprocessing and were adjusted for angle‐specific process physics (Table S1). The LightGBM model then predicted the dimension‐expanded parameters, achieving an angle prediction accuracy exceeding 98%.

**FIGURE 4:**
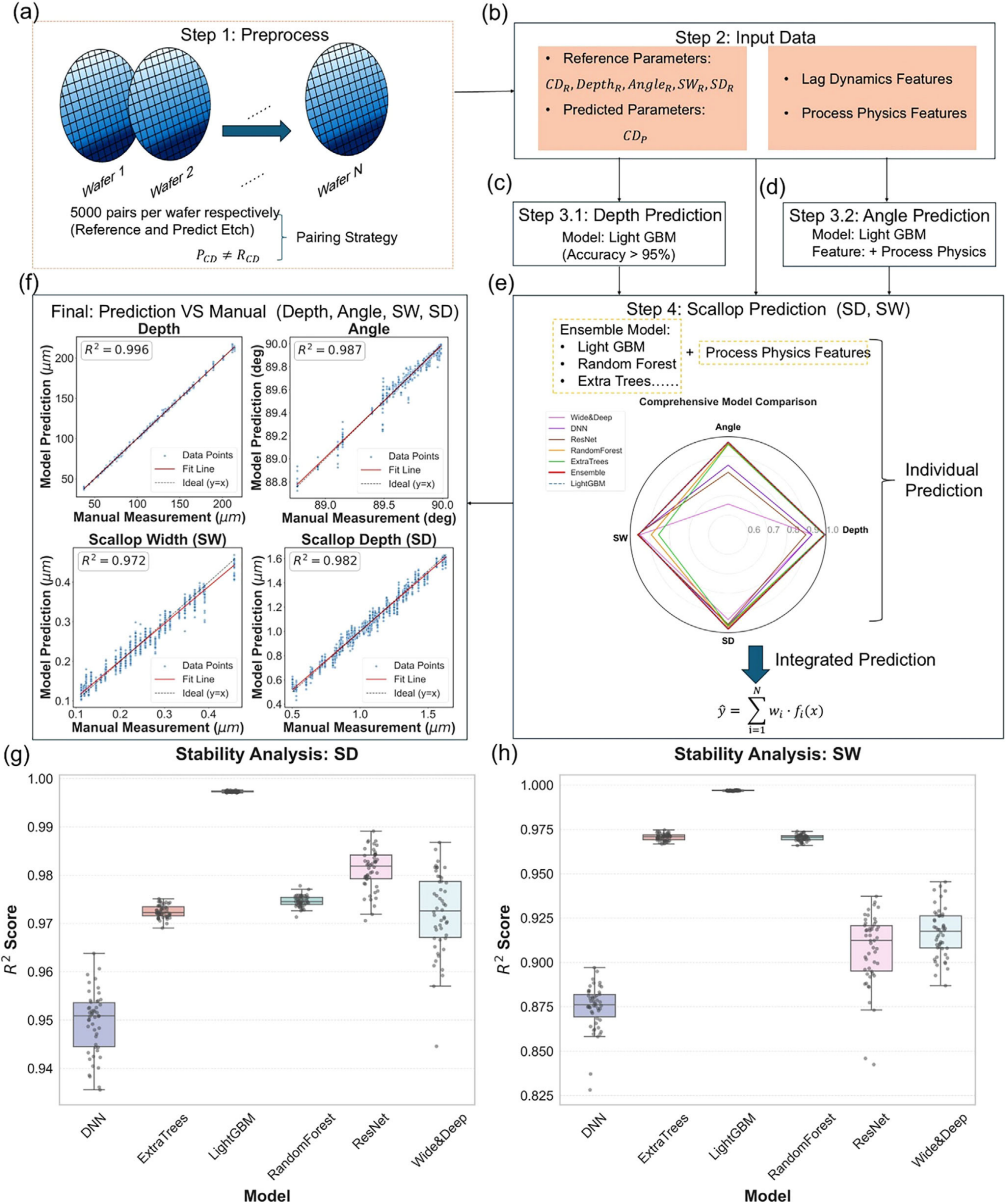
Hybrid ensemble framework and stability validation: (a) data preprocessing in Step 1 of the prediction model; (b) input data and feature dimensions in Step 2 of the prediction model; (c) depth prediction in Step 3.1 of the prediction model; (d) Step 3.2: angle prediction; (e) Step 4: hybrid ensemble model for *SW* and *SD* prediction; (f) prediction results for *Depth*, *Angle*, *SW*, and *SD*; (g) stability analysis of the hybrid ensemble model for *SD*; (h) stability analysis of the hybrid ensemble model for *SW*.

The depth and angle from the previous prediction stage (Step 3) were also fed into the module in Step 4 to predict *SW* and *SD*. Owing to the discrete nature of the Bosch switching cycles, these microfeatures exhibit significant randomness and nonlinearity. Therefore, a hybrid ensemble framework was adopted, incorporating deep learning methods (Wide&Deep, DNN, and ResNet) and tree‐based ensemble learning methods (Random Forest, Extra Trees, and LightGBM). The results are shown in Figure 4e. The individual models demonstrated limited advantages. To enhance the overall model robustness and ensure adaptability to data heterogeneity or quality degradation during future scaling, the hybrid ensemble framework significantly mitigates risks from single‐model bias by integrating the top three performing models (LightGBM, Extra Trees, and DNN). As demonstrated by the maximized area under the radar chart, this hybrid model exhibited superior overall performance to the single baseline models.

The final result ŷ is obtained through the hybrid ensemble framework:

(2) $$\hat{y}=\sum_{i=1}^{N}w_{i}\cdot f_{i}\left(x\right)$$

It is concluded that weights *w_i_* are optimized based on the validation performance of each submodel (visualized in the radar chart). For the current dataset, the radar chart indicates that the results of LightGBM nearly overlap with the curve of the ensemble model. Therefore, *w_i_* for LightGBM in Equation (2) can be set to 1, with all other weights set to 0. For more general models such as those dealing with more complex or lower‐quality datasets, the above weighted approach can be adopted.

The regression plots from the Final Prediction module demonstrate the accuracy of this physics‐based cascading approach. The correlation coefficients were obtained for various physical quantities by comparing the actual data with the model‐generated data. The correlation coefficients were 0.9996 for *Depth*, 0.988 for *Angle*, 0.958 for *SW*, and 0.982 for *SD*, demonstrating that the coupled framework successfully decoupled and reconstructed complex topographies from limited sparse inputs. A mean absolute percentage error (MAPE) analysis comparing the actual and predicted data is presented in Figure S1. The MAPE for *Angle* was only 0.01%, demonstrating exceptional robustness within the HAR range of (88.8°,90.0°). For *Depth*, the MAPE was 0.58%, with the error distribution exhibiting good uniformity across the width range of 50–200 µm. *SD* and *SW* exhibited MAPE values of 2.69% and 4.47%, respectively. Given the small physical dimensions of the scallop patterns, *SD* exhibited isolated outliers approaching 20% error, although the vast majority of data points were clustered within the low‐error range (<5%). Meanwhile, the true values for *SW* were extremely small (0.1–0.45 µm). Even minor absolute deviations are mathematically amplified into large errors. Thus, the observed error primarily stems from the numerical sensitivity of the parameters rather than the insufficient predictive capability. The absence of systematic bias and the fact that samples were predominantly clustered within the low‐error range (<5%) confirm the model robustness, even when capturing high‐frequency microscopic roughness.

To evaluate the robustness of the different models in predicting *SD* and *SW*, stability tests were performed on the six aforementioned architectures (DNN, ExtraTrees, LightGBM, Random Forest, ResNet, and Wide&Deep). The results are shown in Figure 4g,h. Each model underwent 50 independent training iterations using different random seeds, and the distribution of the *R* ^2^ scores on the test set was recorded. Among them, LightGBM demonstrated the highest performance, achieving mean *R* ^2^ values of 0.9973 and 0.9969 for the *SD* and *SW* parameters, respectively, with the corresponding lowest standard deviations of 0.0001 and 0.0002. These results indicate the insensitivity of the model to parameter initialization and its exceptionally high robustness. In contrast, deep learning models, such as ResNet and Wide&Deep, demonstrated competitive performance for *SD* (average *R* ^2^> 0.97) but exhibited high sensitivity to initialization. The standard deviation of ResNet reached 0.0201, which was two orders of magnitude higher than that of LightGBM. However, deep learning models exhibited a steep performance decline in the *SW* prediction task (DNN average *R* ^2^ ≈ 0.87), while tree‐based ensemble models maintained a compact error distribution (average *R* ^2^> 0.97). This highlights the vulnerability of neural networks to local optima under data constraints. Tree‐based models demonstrate superior performance in such scenarios, making them well suited for VM systems that require both high accuracy and reliability in industrial applications.

## 3 Discussion

To objectively validate the accuracy of the predictive model and assess its generalization capability for unknown data, an independent verification wafer was fabricated, and a rigorous cross‐scale validation strategy was implemented. The dataset was stratified based on the top aperture (Top CD) into three categories: Small (S, <15 µm), Medium (M, 15–30 µm), and Large (L, >30 µm). Two challenging inference scenarios, comprising a total of 936 test pairs, were constructed. The first, Small‐to‐Large (S2L), used small‐aperture data to predict large‐aperture profiles, simulating extrapolation from HAR‐constrained environments to open‐field dynamics. The second, Large‐to‐Small (L2S), used large‐aperture data to predict small‐aperture profiles, simulating inversion from transmission‐rich to transmission‐constrained mechanisms. Beyond serving as a generalization test, these two scenarios correspond to distinct practical metrology requirements. The S2L mode can reduce sample preparation time and improve observation efficiency, because small or shallow calibration structures can be sectioned and observed more rapidly and then used to infer larger and deeper HAR structures. In contrast, the L2S mode reduces the technical difficulty of preparing and imaging small‐width HAR structures, for which direct cross‐sectioning, alignment, cleaving, and SEM observation are more challenging.

**FIGURE 5:**
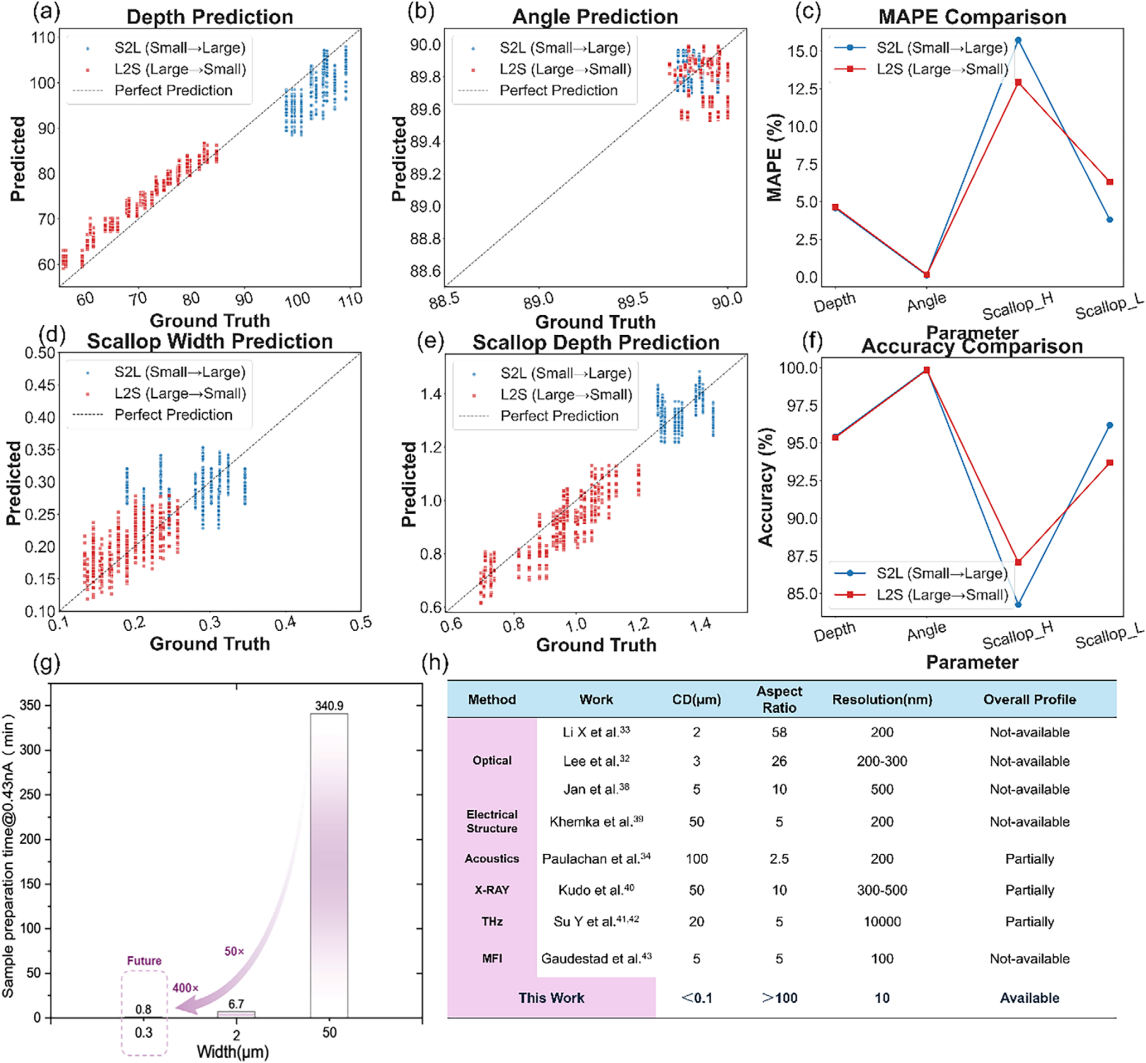
Model generalization capability validation performance test: (a) prediction results of S2L and L2S for *Depth*; (b) prediction results of S2L and L2S for *Angle*; (c) MAPE analysis of S2L and L2S across different physical parameters; (d) prediction results of S2L and L2S for *SW*; (e) prediction results of S2L and L2S for *SD*; (f) accuracy analysis of S2L and L2S predictions across different parameters; (g) efficiency improvement achieved by the proposed model; (h) comparison of observation capabilities of different methods for HAR structures [37, 38, 39, 40, 41].

Figure 5a–e show the regression analyses for *Depth*, *Angle*, *SW*, and *SD*. Figure 5c and f show the prediction accuracy for different physical parameters in the S2L and L2S scenarios, demonstrating the robustness of the model under varying physical constraints. Among the macroscopic features, angle prediction (Figure 5b) achieved near‐perfect accuracy (∼99.9%, MAPE < 0.2%) in both the S2L and L2S scenarios. In Figure 5a, *Depth* prediction maintains a consistent accuracy of ∼95.4% (MAPE ∼4.6%), demonstrating that feature engineering yields minimal error regardless of the prediction direction. Distinct physical discrepancies emerged for the submicroscopic features. Regarding *SD*, the L2S model outperformed S2L (87.1% vs. 84.3%) (Figure 5d). This indicates that the large‐scale prediction approach provides a more stable baseline for roughness estimation. Conversely, for *SW* (Figure 5e), the S2L model achieved higher accuracy (up to 96.2%), whereas the accuracy of the L2S model reached 93.7%. Both models demonstrated high precision and validity. Overall, the deep silicon etching prediction model demonstrated robust bidirectional generalization capabilities, effectively handling the complex nonlinearities of Bosch deep silicon etching across different scaling mechanisms. In contrast, the predictive accuracy of conventional data‐driven models typically degrades rapidly outside the training aperture range owing to unmodeled ARDE and transport‐limited effects.

Figure 5g shows the FIB cross‐sectional observation time for samples with varying widths under a constant beam current and identical etching conditions. As the sample width decreased, the preparation time was significantly shortened. Specifically, when the sample width decreased from 50 to 2 µm, the preparation time decreased to 1/50 of the original duration, exhibiting a nearly exponential relationship. Therefore, the cross‐sectional observation efficiency can be effectively enhanced by utilizing the proposed AI model to place small‐line‐width patterns at specific locations on the wafer and predict the characteristics of larger, deeper patterns in other regions. Furthermore, as the observations are confined to the shallow surface layer of the wafer, the impact on subsequent wafer processes is minimized. This strategy helps reduce observation‐related damage costs during wafer manufacturing. The chart data also indicate that as the linewidths continue to shrink, the predictive model can further enhance the FIB process efficiency, with the potential to reduce the preparation time to 1/400 of the original.

Figure 5h presents benchmark comparisons of the proposed methodology against established metrology paradigms for characterizing HAR semiconductor structures. Conventional optical methods (e.g., those of Li et al., and Thuy et al.,) are fundamentally constrained by the diffraction limit and depth‐of‐field restrictions, limiting their efficacy to lower aspect ratios (<60:1) and relatively coarse critical dimensions (*CD* > 2 µm). Although penetrative techniques, such as acoustic, X‐ray, and terahertz imaging, extend detection capabilities to subsurface regions, they suffer from insufficient spatial resolution (typically 200–500 nm) and, critically, fail to reconstruct the complete sidewall morphology. The proposed method overcomes these limitations by bridging the gap between macroscopic depth penetration and microscopic feature fidelity. The proposed approach achieves a breakthrough spatial resolution of 10 nm, an order‐of‐magnitude improvement over the best‐performing metrology‐by‐feature inference methods, while accommodating ultra‐scaled dimensions (*CD* < 0.1 µm) and extreme aspect ratios (>100:1). Most importantly, in contrast to prior approaches that yield only discrete metrics, the proposed method provides a holistic, continuously resolved profile, enabling comprehensive visualization of the full deep‐trench geometry. This capability is pivotal for next‐generation process control, where the detection of submicrometer‐scale defects in ultradeep structures is required but remains challenging with existing techniques.

## 4 Conclusion

In this study, a VM framework was developed for deep silicon etching that integrates physical mechanisms with AI. The framework establishes accurate mapping from 2D surface observation data to the full 3D morphology of HAR structures, overcoming the resolution limitations of traditional indirect measurement methods and the efficiency bottlenecks of destructive sampling. By integrating a subpixel feature extraction network based on the YOLO‐Pose architecture with a hybrid ensemble model and incorporating fundamental plasma‐etching physics, the proposed framework effectively decouples the nonlinear characteristics of the Bosch process from sparse surface observations. It achieves nondestructive reconstruction of morphological parameters, including the *Depth*, sidewall inclination, and microscopic scalloping, with an average accuracy of 93.96%. In particular, the bidirectional cross‐scale prediction strategy provides two complementary process advantages: S2L prediction improves observation efficiency by reducing sectioning time for large and deep HAR features, whereas L2S prediction reduces the preparation and imaging difficulty of small‐width HAR structures. Rigorous multiscale validation confirmed the strong generalization capability of the model across varying pattern densities and demonstrated a significant reduction in the data acquisition time from hours (FIB/SEM sectioning) to <1 min. As an enabling technology for next‐generation autonomous semiconductor manufacturing, this physics‐driven AI methodology provides near‐real‐time process feedback as an alternative to traditional destructive inspection. Future research will involve extending this framework to diverse etching chemistries and multilayer device stacks and incorporating in situ plasma and process signals to enable closed‐loop, real‐time process control.

## 5 Methods

### 5.1 Sample Fabrication

Experiments were conducted using 8‐inch (200‐mm) P100 wafers with an initial thickness of 725 µm and resistivity controlled within the range of 8–12 Ω·cm. First, the AZ6130 photoresist was spin‐coated at 2000 rpm and pre‐baked at 110°C for 90 s to form a photoresist film with a thickness of ∼3.8 µm. Pattern transfer was completed using an MA8 lithography system in the hard contact mode with an exposure energy of 330 mJ. The mask integrated pattern arrays with varying line widths (2–50 µm) and densities (1%–84%) for cross‐validation. The corresponding parameters are listed in Table S1. Post‐lithography processing involved developing patterns in the MIF300 developer at room temperature for 60 s, followed by curing at 110°C for 2 min to complete pattern formation. Etching was then performed using an SPTS‐Omega LPX etch system under the following process conditions: SF_6_ at 700 sccm, C_4_F_8_ at 300 sccm, source RF power of 2500 W, and bias RF power of 150–250 W. To reduce the risk of model overfitting caused by excessive concentration in the training data distribution, three etching cycle counts (50, 100, and 150 loops) were applied to different wafers to generate data samples with diverse topographical features. After etching, the wafers were cut along the pattern centerline using the DSI‐TC9211 laser invisible cutting system. To prevent laser cutting from affecting the morphology of the target cross‐section, the laser focus position was set to a depth below the etching depth, ensuring that the modified layer was formed beneath the target structure. Subsequently, the cut specimens were cleaved using the DSI‐L‐1126 fully automated cleaving system, and the Zeiss Gemini 300 system was employed for cross‐sectional morphology imaging and data acquisition, thereby completing the sample preparation and data acquisition process.

### 5.2 Topological Feature Extraction

To quantify the complex etching morphology nondestructively, raw cross‐sectional SEM images were first processed using a customized deep CNN based on the YOLO‐Pose architecture. In this process, a representative subset of the dataset was manually annotated using a rigorous five‐point topological markup protocol to define the geometric ground truth, replacing traditional bounding boxes with precise keypoint semantics. The network employed PANet to fuse multiscale features extracted by the backbone, effectively handling significant aspect‐ratio variations inherent in silicon‐etched structures. The detection head subsequently regressed the subpixel coordinates of the critical geometric keypoints, specifically the Top CD, Bottom CD, and *Depth*, directly from the pixel intensity gradients, enabling end‐to‐end automated metrology with micrometer‐level precision. The YOLO‐Pose visual encoder was initialized from the pretrained YOLO11s‐pose weights and trained for 100 epochs with an input image size of 640 × 640 pixels, a batch size of four, and an initial learning rate *l* *r*_0_ =  0.01 with a final learning‐rate factor *lr_f_* = 0.01. The optimizer was set to the Ultralytics automatic optimizer selection mode, with momentum = 0.937, weight decay = 0.0005, three warm‐up epochs, early‐stopping patience of 20 epochs, mixed‐precision training enabled, and a random seed of zero. The annotated dataset used for YOLO‐Pose training was split into training and validation subsets at an 80:20 ratio.

### 5.3 Hybrid Ensemble Model

To establish a predictive framework for *SW* and *SD* parameters and validate the model stability for ensuring generalization capability across varying initialization conditions, six distinct model architectures were constructed for systematic stochastic stability analysis. These models were categorized into two primary classes: tree‐based ensemble methods (LightGBM, Random Forest, and Extra Trees) and deep learning architectures (DNN, ResNet, and WideDeep). Deep learning configurations, such as the DNN, were constructed as a three‐layer perceptron (128–256–128 neurons), incorporating Batch Normalization and Dropout layers (dropout probability *p*  =  0.2) to mitigate internal covariate shifts and suppress overfitting. ResNet addresses the degradation problem in deep networks using an architecture comprising two residual blocks, each containing 128 hidden neurons. Wide&Deep was designed to jointly train a wide linear model for memorizing sparse feature interactions and a deep feedforward network (64–32 neurons) for generalizing dense embedding features. For the deep learning models used in the stochastic stability analysis, all neural networks were trained from scratch using the Adam optimizer and mean squared error loss. The learning rate was set to 0.001, the batch size was 256, and each model was trained for 50 epochs for each random seed. Input features were standardized using z‐score normalization before model training. The DNN consisted of fully connected layers with 128–256–128 hidden neurons, Batch Normalization, Rectified Linear Unit activation, and a dropout probability of 0.2. The ResNet contained two residual blocks with 128 hidden neurons and a dropout probability of 0.1 within each block. The Wide&Deep model combined a linear wide branch with a deep branch of 64–32 hidden neurons.

Additionally, stability analysis was conducted using Monte Carlo cross‐validation. The experimental protocol involved the execution of 50 independent trials (*N*  =  50) for each architecture, with the pseudorandom number generator seed sequentially adjusted from 0 to 49. In each iteration, the seed controlled two critical stochastic factors: the random partitioning of the dataset into training (80%) and test (20%) sets. The model parameters (e.g., neural‐network weights and decision‐tree splitting criteria) were initialized. For each trial, the model was retrained from scratch, and the coefficient of determination (*R* ^2^) was recorded. The statistical distribution (mean and standard deviation) of these 50 performance metrics served as the core criterion for evaluating stability. This approach ensured that the observed predictive superiority stemmed from the intrinsic architecture of the proposed framework rather than being an artifact of specific random initializations or data partitions.

## Author Contributions

**Shuyan He**: software, writing – original draft, writing – review and editing. **Wei Wang**: funding acquisition, writing – review and editing, supervision. **Lang Chen**: writing – original draft, data curation, conceptualization, methodology, formal analysis. **Yufeng Jin**: writing – review and editing, validation, supervision, project administration. **Bo Wen**: software, methodology, visualization, data curation, supervision, writing – original draft, writing – review and editing, conceptualization.

## Conflicts of Interest

The authors declare no conflicts of interest.

## Supporting information




**Supporting File**: advs76723‐sup‐0001‐SuppMat.docx.

## Data Availability

The data that support the findings of this study are available from the corresponding author upon reasonable request.

## ENDNOTE

1 Encyclopedia of Microfluidics and Nanofluidics
