# Supplementary material for: Pattern‐Aware Intelligence Enables Nondestructive, Rapid Quantification of High‐Aspect‐Ratio Silicon Etching
Source: Adv Sci (Weinh). 2026 Jul 20:e76723. Online ahead of print. doi: 10.1002/advs.76723 (PMC13383699; doi:10.1002/advs.76723)
Supplement: Supplementary file 1 — Supporting File: advs76723‐sup‐0001‐SuppMat.docx. [file ADVS-9999-e76723-s001.docx]

**Feature Descriptor Construction and Model Symbols**

To quantify the geometric boundary conditions governing ion flux and etchant diffusion, we constructed a set of transport descriptors. The Reference Aspect Ratio ($AR_{R}$) is established as the fundamental baseline for characterizing Aspect Ratio Dependent Etching (ARDE), directly representing the impedance to species transport within high-aspect-ratio trenches. To model the scaling laws between the reference and target structures, we introduced the CD Ratio (${CD}_{ratio}$) and CD Delta ($\Delta CD$). These variables capture the non-linear modulation of the etch rate induced by the micro-loading effect and pattern density variations. Furthermore, to account for the diffusion hindrance in sub-micron apertures, the Target CD Squared (${CD}_{P_{sq}}$) and Inverse Aperture (${CD}_{P_{inv}}$) are incorporated, modeling the inverse relationship between opening size and gas conductance in the Knudsen transport regime. Finally, the Lag Factor (${Lag}_{factor}$) and Aspect Ratio Interaction (${AR}_{interaction}$) couple the vertical depth with lateral scaling dimensions, resolving the synergistic impact of RIE lag and geometric shadowing on the evolving etch front.

To decode the cyclic dynamics inherent to the Bosch process, micro-morphological features were derived to characterize the sidewall periodicity. The Scallop Aspect Ratio (${AR}_{R_{sca}}$) and Scallop Area (${Area}_{R_{sca}}$) serve as morphological fingerprints, elucidating the competition between the isotropic etching phase and the passivation deposition phase. Complementing these are frequency-domain descriptors, Scallop Density (${Density}_{R_{sca}}$) and Cycle Count ($N_{cycle}$), which correlate the spatial frequency of the sidewall roughness with the temporal switching frequency of the plasma reactor. To normalize these local perturbations against global dimensions, we utilized Normalized Scallop Width ($w_{norm}$) and Normalized Scallop Depth ($l_{norm}$), quantifying the relative impact of sidewall roughness on the critical dimension. Additionally, Global Width Ratio ($\gamma_{w}$) and Global Depth Ratio ($\gamma_{d}$) decouple the stochastic fluctuations of individual cycles from the cumulative etch depth, allowing the model to distinguish between depth-dependent process drift and local kinetic instability.

To resolve high-order non-linearities, the model architecture integrates advanced interaction terms and a hierarchical cascade strategy. Shape Interaction ($\varphi_{shape}$) and Density Interaction ($\varphi_{dens}$) are synthesized to model the cross-coupling effects where lateral aperture constraints modulate the localized scallop morphology. Geometrically, the angular data is transformed into Reference Slope ($tan\theta_{ref}$) and Predicted Slope ($tan\theta_{pre}$) using tangent functions, linearizing the gradient space to better suit regression convergence near vertical profiles. Crucially, the system employs a causality-driven cascade mechanism: predictions of global geometry—Predicted Depth ($\hat{Angle}$), Predicted Aspect Ratio ($\hat{AR}$), and Predicted Angle ($\hat{Angle}$)—are injected as latent state priors into downstream models. This hierarchical approach mimics the physical reality where the formation of local fine-grained features ($SW, SD$) is conditionally dependent on the instantaneous global geometric state of the evolving trench.

Table S1: Feature definitions

| Feature Category | Feature Name | Definition |
| --- | --- | --- |
| Transport Constraints | $AR_{R}$ | $\frac{{Depth}_{R}}{CD_{R}}$ |
|  | ${CD}_{ratio}$ | $\frac{CD_{P}}{CD_{R}}$ |
|  | $\Delta CD$ | $P_{CD}-R_{CD}$ |
|  | ${CD}_{P_{sq}}$ | ${CD}_{P}^{2}$ |
|  | ${CD}_{P_{inv}}$ | $\frac{1}{CD_{P}}$ |
|  | ${Lag}_{factor}$ | $Depth_{R}\times{CD}_{ratio}$ |
|  | ${AR}_{interaction}$ | $AR_{R}\times{CD}_{ratio}$ |
| Scallop Features | ${AR}_{R_{sca}}$ | $\frac{{SD}_{R}}{SW_{R}}$ |
|  | ${Density}_{R_{sca}}$ | $\frac{{SD}_{R}}{SW_{R}}$ |
|  | ${Area}_{R_{sca}}$ | ${SD}_{R}\times SW_{R}$ |
|  | $w_{norm}$ | $\frac{{SW}_{R}}{{CD}_{R}}$ |
|  | $l_{norm}$ | $\frac{{SD}_{R}}{{CD}_{R}}$ |
|  | $\gamma_{w}$ | $\frac{{SW}_{R}}{{Depth}_{R}}$ |
|  | $\gamma_{d}$ | $\frac{{SD}_{R}}{{Depth}_{R}}$ |
|  | $N_{cycle}$ | $\frac{{Depth}_{R}}{{SD}_{R}}$ |
|  | $\varphi_{shape}$ | ${CD}_{ratio}\times{AR}_{R_{sca}}$ |
|  | $\varphi_{dens}$ | $\Delta CD\times{Density}_{R_{sca}}$ |
| Angle Transformation Features | $tan\theta_{ref}$ | $tan(radians(\theta_{R}))$ |
|  | $tan\theta_{pre}$ | $tan(radians(\theta_{P}))$ |
| Cascade Prediction Features | $\hat{AR}$ | $\frac{\hat{Depth}}{CD_{T}}$ |
|  | $\hat{Depth}$ | $Model_{Depth}.predict()$ |
|  | $\hat{Angle}$ | $Model_{Angle}.predict()$ |

Table S II: Nomenclature of etch profile geometric parameters and model symbols

| Symbol | Definition |
| --- | --- |
| $CD$($\mu m$) | The width of etch structure. |
| $w_{top}/CD_{top}$ | The top width of etch structure. |
| $w_{bottom}$ | The bottom width of etch structure. |
| $Depth$ | The depth of etch structure. |
| $\theta$ | Inclination of the trench sidewall. |
| $SW$ | The lateral depth of periodic ripples on sidewalls formed by the Bosch process. |
| $SD$ | The vertical height of a single scallop cycle on the sidewall. |
| $R/P$ | $R$ for shallow/known regions (reference) and $P$ for deep/unknown regions (predicted). |
| $\hat{y}$ | The final synthesized prediction value obtained via the weighted ensemble formula as ensemble output. |
| $X_{abs}$ | The absolute geometric feature vector extracted by the YOLO-Pose network: [$CD, Depth, \theta$] |

**Error Analysis of YOLO Model Results**

To rigorously assess the inference robustness across the comprehensive feature space, the Absolute Percentage Error (APE) distributions for four key morphological parameters were analyzed against ground truth measurements. The results reveal a distinct hierarchical precision correlated with the physical scale of the features. For the macroscopic governing parameters, the model demonstrates near-deterministic convergence. As illustrated in Fig. S1(a), the Sidewall Angle achieves a vanishingly low Mean Absolute Percentage Error (MAPE) of 0.01%, with error distributions tightly compressed near the zero-deviation line, confirming that the ion sheath trajectory and resulting profile directionality are captured with high stability independent of pattern density. Similarly, the Etch Depth (Fig. S1(b)) maintains a robust MAPE of 0.58% across a broad dynamic range (30–220$\mu m$), indicating that the physics-informed feature space successfully linearizes the aspect-ratio-dependent etching (ARDE) lag effects without systematic bias.

In contrast, the microscopic morphological descriptors—Scallop Depth (Fig. S1(c)) and Side Wall Roughness (Fig. S1(d))—display slightly elevated variances, with MAPEs of 2.69% and 4.47%, respectively. Notably, these distributions exhibit distinct heteroscedasticity, where percentage errors amplify significantly as feature sizes decrease toward the sub-micron regime (SW < 0.2$\mu m$). This phenomenon is attributed to the inherent stochasticity of the Bosch process switching cycles and the resolution limits of metrology at these scales. Consequently, the global error remains well within the acceptable tolerance for semiconductor process control, validating the model's capability to reconstruct complex hierarchical topologies.


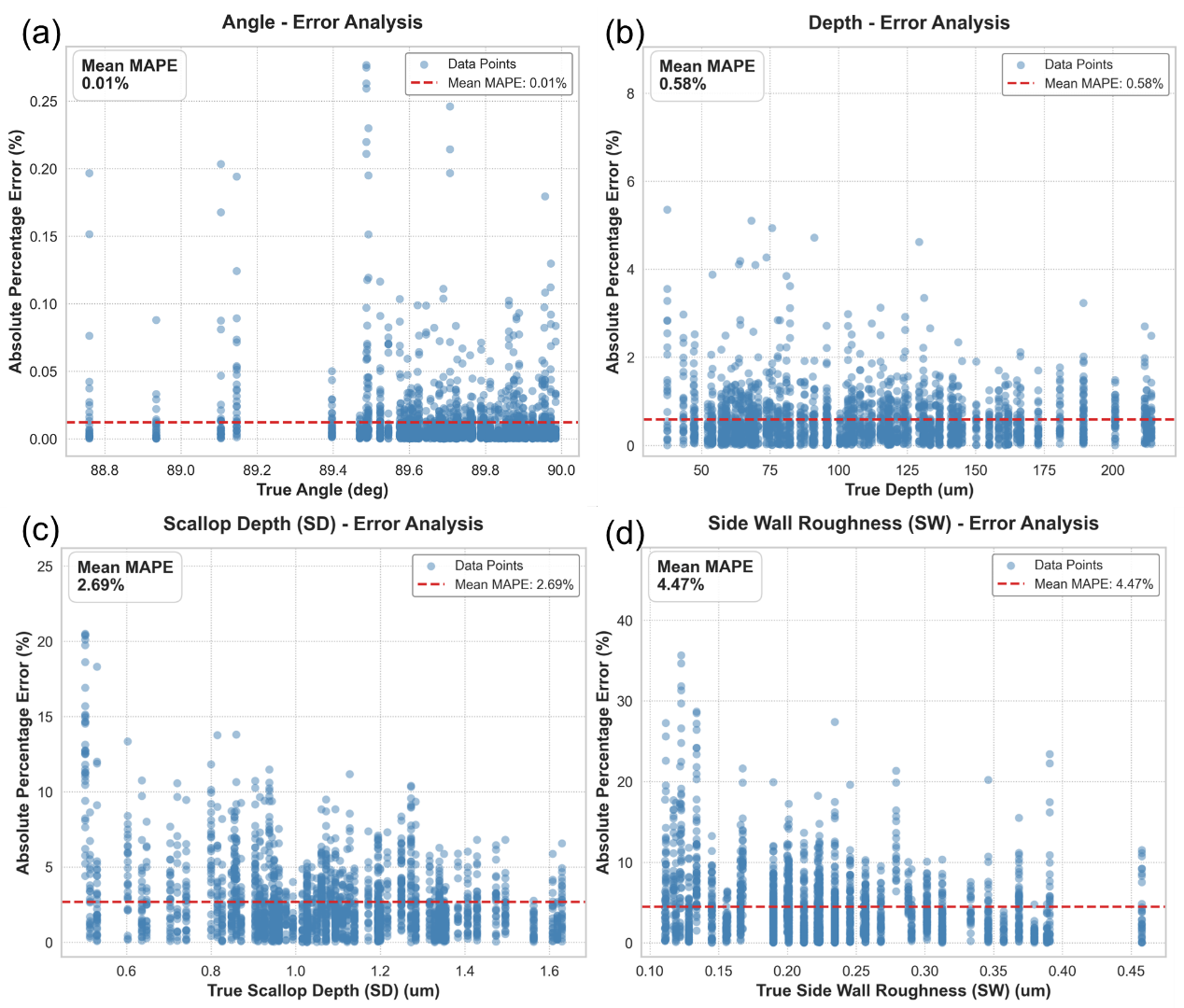


Figure S1. Quantitative assessment of predictive fidelity across macroscopic and microscopic morphological scales. (a) error analysis of $Angle$ parameter; (b) error analysis of $Depth$ parameter; (c) error analysis of $SD$ parameter; (d) error analysis of $SW$ parameter.
